# A Dynamic Analysis of Toron Formation in Chiral Nematic Liquid Crystals Using a Polarization Holographic Microscope

**DOI:** 10.3390/polym17131849

**Published:** 2025-07-02

**Authors:** Tikhon V. Reztsov, Aleksey V. Chernykh, Tetiana Orlova, Nikolay V. Petrov

**Affiliations:** 1 Research and Educational Center for Photonics and Optical IT, ITMO University, 3 Kadetskaya Liniya V.O., bld. 2, lit. A, Saint Petersburg 197101, Russia; chernykh_a@itmo.ru (A.V.C.); n.petrov@niuitmo.ru (N.V.P.); 2 Infochemistry Scientific Center, ITMO University, 9 Lomonosova Str., Saint Petersburg 197101, Russia; tetiana.orlova@ysu.am; 3 Institute of Physics, Yerevan State University, Yerevan 0025, Armenia; 4 School of Physics, Harbin Institute of Technology, Harbin 150001, China

**Keywords:** phase modulation, digital polarization holography, liquid crystal, chiral nematics, topological soliton, toron

## Abstract

Topological orientation structures in chiral nematic liquid crystals, such as torons, exhibit promising optical properties and are of increasing interest for applications in photonic devices. However, despite this attention, their polarization and phase dynamics during formation remain insufficiently explored. In this work, we investigate the dynamic optical response of a toron generated by focused femtosecond infrared laser pulses. A custom-designed polarization holographic microscope is employed to simultaneously record four polarization-resolved interferograms in a single exposure. This enables the real-time reconstruction of the Jones matrix, providing a complete description of the local polarization transformation introduced by the formation of the topological structure. The study demonstrates that torons can facilitate spin–orbit coupling of light in a manner analogous to q-plates, highlighting their potential for advanced vector beam shaping and topological photonics applications.

## 1. Introduction

Liquid crystals (LCs) have become key components in modern photonics and display technologies due to their tunable optical properties, high birefringence, and strong response to external stimuli [1,2]. These properties make them especially attractive for applications in adaptive optics, optical sensing, and reconfigurable beam shaping [3]. Among the various LC phases, chiral nematic LCs are of particular interest due to their ability to host topological solitons [4], which are localized and metastable three-dimensional twisted configurations of the director field. In this context, torons, or triple-twisted elastic excitations, are a well-studied example of topological solitons in chiral nematics [5,6,7]. The topological structure of a toron comprises a double-twist cylinder surrounded by two hyperbolic point defects [5]. For example, torons have been explored as reconfigurable lenses [8], tunable diffraction gratings, and vortex beam generators [9,10], as well as polarization-controlled vector beam shapers and integrated topological elements inside optical resonators [11,12]. This versatility makes torons an important platform for developing advanced spin–orbit photonic devices and dynamic topological optics.

In addition, photopolymerization of liquid crystals has been recognized as a powerful method for stabilizing complex orientational structures and fabricating mechanically robust LC-based photonic elements. By incorporating reactive mesogens into chiral nematic mixtures and selectively curing them with ultraviolet (UV) light, it is possible to preserve topological configurations such as torons or cholesteric fingers, along with their corresponding optical responses and director field patterns [13]. This technique enables not only the fabrication of reconfigurable and tunable diffractive optical elements but also the layer-by-layer construction of three-dimensional defect lattices with high structural fidelity. Moreover, photopolymerized LC templates can function as scaffolds for the directed self-assembly of nanoparticles, thereby unlocking new possibilities in nanophotonics, metamaterials, and light–matter interaction for energy applications [14]. The ability to convert dynamic LC configurations into solid-state architectures highlights the critical role of photopolymerization in linking soft-matter physics to practical device technologies.

However, despite growing interest in the development of topological optical elements and their implementation in photonic devices and circuits, the dynamics of toron formation under laser excitation remain poorly understood [15]. Previous studies have primarily focused on the optical generation and recording of torons and other topological solitons using structured light, with limited attention paid to the real-time processes governing their nucleation and evolution. Understanding these dynamics is essential for the precise fabrication of diffractive optical elements through controlled laser structuring.

Conventional optical characterization techniques often fail to resolve the full complexity of topologically rich and anisotropic LC configurations, especially under dynamic conditions [16,17]. Although digital holography has been widely used to obtain information about optical fields [18], its application to anisotropic and birefringent media such as LCs remains challenging due to polarization-dependent distortions [19,20]. Moreover, standard digital holographic microscopy techniques generally rely on static or quasi-static conditions, limiting their utility for observing rapidly evolving optical phenomena such as toron formation [12,21]. Extensive research has been conducted on polarization measurement techniques in the field of holography [22,23,24,25,26,27]. For instance, several recent approaches aim to reconstruct the full Jones matrix of anisotropic samples in a single shot, leveraging polarization multiplexing and spatial carrier encoding. In [28], a polarization digital holographic microscope employs an interferometer with tunable spatial carriers to retrieve polarization-resolved holograms using standard sensors, such as those based on charge-coupled devices. In [29], the authors utilize a four-channel digital polarization holography system that combines a Ronchi grating with a spatial light modulator to spatially separate all four Jones matrix elements in the Fourier domain. Other notable systems include compact lensless architectures [30], polarization gratings [31], and quasi-common-path interferometers designed for real-time birefringence mapping [32]. Although these methods demonstrate impressive performance in stability, compactness, or phase sensitivity, they often involve compromises. The system in [33] requires two polarization measurements despite its near-single-shot capability, while [34] maintains a single-exposure operation but uses scanning for enhanced sensitivity, and [35] relies on active polarization modulation using electrically controlled components, which introduces potential stability issues. In [36], the authors demonstrate a compact in-line configuration with a Wollaston prism and beam displacer, using iterative reconstruction to extract polarization information from polarization-multiplexed holograms. Similar to the approach in [37], this method also relies on spatial filtering and iterative algorithms.

In this context, we present a polarization holographic microscope (PHM) that combines an off-axis Mach–Zehnder interferometric configuration with a polarization-sensitive camera. This setup enables the single-shot acquisition of four polarization-resolved interferograms and the reconstruction of the full Jones matrix. Unlike polarization holography methods based on spectral separation or computational demosaicing, our setup provides a complete description of both the amplitude–phase and polarization characteristics of the light field without requiring iterative processing. In addition, the system benefits from a simple design in which both reference beams propagate along a common optical path. This geometry ensures high stability of the polarization measurements. Our system is particularly suited for studying the dynamic light–matter interactions in transparent media, such as the laser-induced formation of torons in chiral nematic LCs. We integrate this system with a femtosecond infrared laser that enables controlled local heating of the LC cell, inducing toron formation. The ability to monitor the formation of torons in real time provides insight into the physical mechanisms underlying topological transitions in chiral nematic LCs. We demonstrate that torons affect not only the spatial phase and polarization distribution of transmitted light but also act as functional optical elements. The presented results show that the PHM is a powerful tool for exploring light–matter interaction in soft topological media, with promising implications for tunable photonic systems, including spin–orbit converters, reconfigurable waveplates, and polarization-controlled beam-shaping devices.

## 2. Materials and Methods

### 2.1. LC Samples

In this study, we used a chiral liquid crystal mixture based on an E7-like nematic LC (HPC850600-100, HCCH, Beijing, China) doped with the chiral additive S811 (HCCH, Beijing, China, CAS 87321-20-8) at a concentration of C=0.82wt.%. The left-handed additive S811 is a standard, widely used, and commercially available additive, which caused its selection in our case. The use of a chiral dopant is necessary to induce a chiral nematic (cholesteric) phase, the frustrated state of which precisely hosts topological solitons such as torons.

The frustration of the cholesteric helix is achieved in plane LC cells with homeotropic boundary conditions when the helical pitch exceeds the critical pitch value determined by the cell thickness and the elastic constants of the LC. Taking the E7 parameters, this critical pitch is approximately 11 μm for a 10 μm-thick LC cell. Such pitch values, on the order of tens of microns, are usually obtained by doping an achiral nematic LC with chiral organic molecules. The cholesteric pitch was measured using the Grandjean–Cano wedge method and was found to be approximately 11 μm. The refractive indices of the pure nematic LC are $n_{e}$ = 1.741 and $n_{o}$ = 1.517, and the phase transition from the nematic to the isotropic LC state occurs at +59 ^∘^C.

**Figure 1:**
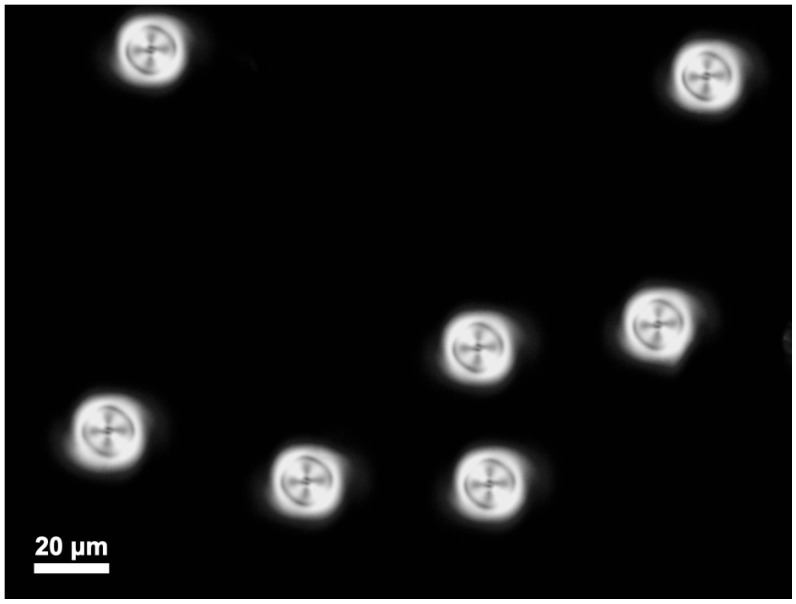
Torons formed in a frustrated chiral LC film (E7 + S811). The image was taken under crossed polarizer imaging conditions.

The chiral LC mixture was filled into a 10 μm-thick homeotropic ITO-coated commercial LC cell (E.H.C., Tokyo, Japan). Perpendicular molecular alignment on both substrates, combined with the given ratio of the cholesteric pitch to the cell thickness, ensured the frustration of the cholesteric helix. After localized exposure to a light beam, the cholesteric helix locally returns to its twisted state, and long-lived localized orientational structures are formed, identified by their appearance in cross-polarized optical microscopy images as torons (Figure 1) [38]. Their transverse size was approximately equal to the thickness of LC film, which was consistent with previously reported data on torons [5,39].

### 2.2. PHM Setup

**Figure 2:**
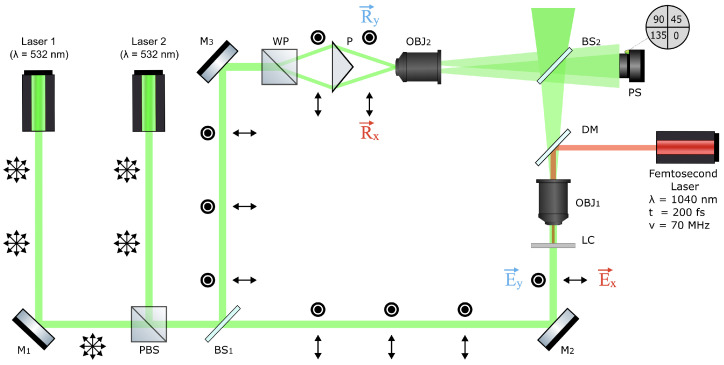
Basic optical scheme of the PHM. DM: dichroic mirror; BS: beam splitter; M: mirror; PBS: polarizing beam splitter; P: prism; WP: Wollaston prism; OBJ: objective; LC: liquid crystal; PS: polarizing sensor.

The PHM is designed to analyze the dynamic evolution of anisotropic polarization and amplitude–phase characteristics in transparent samples. Our setup is based on an off-axis Mach–Zehnder interferometric layout with two identical 40× microscope objectives (OBJ_1_ and OBJ_2_), as shown in Figure 2. This design minimizes the impact of low-frequency spatial distortions that commonly affect interferometric setups, improving the fidelity of measurements in complex optical environments. Enhanced stability against mechanical vibrations and reliable interference of mutually incoherent beams are ensured by propagating both reference beams through identical optical elements in the second arm of the interferometer.

The PHM utilizes two 532 nm laser modules as probe beams, which are initially randomly polarized. A non-polarizing beam splitter (BS) divides the beam into object and reference arms, which are directed using mirrors (M) positioned to define the optical paths. A polarizing beam splitter (PBS) further separates each beam into two orthogonally polarized components, horizontally and vertically polarized.

To distinguish the resulting interference patterns from each beam, the reference arm incorporates a Wollaston prism (WP), which splits the reference beam into two orthogonally polarized components without significant intensity loss. These beams are directed at different angles into the reference objective using a standard prism (P) and are later recombined inside the imaging path using a standard prism. A crucial feature of our implementation is the use of two reference beams with orthogonal polarization states, eliminating the need for mechanical polarization rotation (e.g., with a half-wave plate).

The first objective (OBJ_1_) in the object arm of the interferometer transfers the magnified image of the sample to the matrix detector plane. Similarly, the second objective (OBJ_2_) in the reference arm works in an analogous way, translating the two inclined wavefronts from the two sources. The interference field is recorded on the polarization sensor (PS) camera (FLIR BFS-U3-51S5P-C), which plays a crucial role in accurately capturing polarization information during the experiment [14,29,35,40,41]. The acquired images are processed using a Fourier spectrum-based algorithm combined with Jones formalism to extract polarization characteristics in real time.

To observe the impact of femtosecond infrared (IR) illumination on the formation of torons in the LC sample, a dichroic mirror (DM) was incorporated into the object arm of the PHM setup. The IR source employed in the experiment was a femtosecond laser system (SOLAR Laser Systems, Minsk, Belarus, model FL-1), operating at a central wavelength of 1040 nm with an average output power of approximately 1 W. The laser emitted pulses with an energy of 15 nJ, a duration of 200 fs, and a repetition rate of 70 MHz. For clarity, collimation optics and neutral density filters are omitted from the schematic. The attenuated IR beam was directed through OBJ_1_ and focused into a 5 μm spot with a total power of 50 mW. Within this focal region, local heating of the LC sample was induced. The 1040 nm wavelength was selected based on the availability of stable femtosecond laser systems in this range. E7 nematic liquid crystals are known to be nearly transparent at 1040 nm [42], which allows localized energy delivery into the sample without significant heating outside the laser focus. This ensures controlled toron generation with minimal disturbance to the surrounding regions.

A key advantage of our PHM system is its ability to perform continuous polarization-resolved imaging, enabling real-time tracking of toron formation and transformation. This capability provides new insights into topological defects and optical phenomena in liquid crystals, significantly advancing the field of polarization microscopy.

### 2.3. Method of the Vector Field Reconstruction

**Figure 3:**
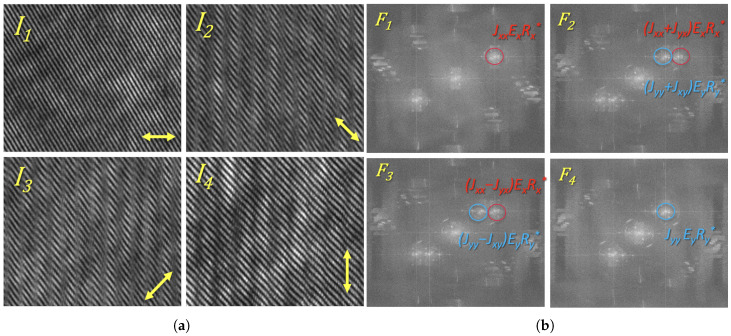
Single-shot reconstruction of the Jones matrix. (**a**) Magnified and separated interferograms of the optical field, corresponding to four polarization-resolved intensity projections recorded by the polarization camera. (**b**) Amplitude distribution of the Fourier spectrum for each projection shown in (**a**), illustrating the spatial frequency components associated with the individual elements of the Jones matrix.

The unique design of the developed PHM system enables the simultaneous capture of all components of the optical vector field in a single exposure. A key component of the setup is the polarization matrix sensor, which records four distinct polarization-resolved intensity projections corresponding to linear polarization orientations at 0°, 45°, 90°, and 135°. This allows us to register four digital holograms in parallel, as illustrated in Figure 3a.

The object beam encodes the polarization-modified field transmitted through the LC sample. Specifically, horizontal ($E_{x}$) polarization components are present in holograms $I_{1}$, $I_{2}$, and $I_{3}$, while vertical ($E_{y}$) components are found in $I_{2}$, $I_{3}$, and $I_{4}$ (as indicated in Figure 3b).

The resulting four-beam interference field is described by the following expression:

(1) $$E=\left(\begin{array}{cc} J_{xx} & J_{xy}\\ J_{yx} & J_{yy} \end{array}\right)\left(\left(\begin{array}{c} E_{x}\\ 0 \end{array}\right)+\left(\begin{array}{c} 0\\ E_{y} \end{array}\right)\right)+\left(\begin{array}{c} R_{x}\\ 0 \end{array}\right)+\left(\begin{array}{c} 0\\ R_{y} \end{array}\right),$$

where

- $\left(\begin{array}{cc} J_{xx} & J_{xy}\\ J_{yx} & J_{yy} \end{array}\right)$ is the Jones matrix of the LC sample;
- $\left(\begin{array}{c} E_{x}\\ 0 \end{array}\right)$ and $\left(\begin{array}{c} 0\\ E_{y} \end{array}\right)$ are two noninterfered illuminating objects beams;
- $\left(\begin{array}{c} R_{x}\\ 0 \end{array}\right)$ and $\left(\begin{array}{c} 0\\ R_{y} \end{array}\right)$ are corresponding reference beams.

Each of the four recorded intensity projections ($I_{1}$ to $I_{4}$) corresponds to a specific interference pattern between components of the object and reference fields:

(2) $$\begin{array}{cc} I_{1} & ={\left|J_{xx}E_{x}+J_{xy}E_{y}+R_{x}\right|}^{2}, \end{array}$$

(3) $$\begin{array}{cc} I_{2} & =\frac{1}{2}{\left|\left(J_{xx}+J_{yx}\right)E_{x}+\left(J_{xy}+J_{yy}\right)E_{y}+R_{x}+R_{y}\right|}^{2}, \end{array}$$

(4) $$\begin{array}{cc} I_{3} & =\frac{1}{2}{\left|\left(J_{xx}-J_{yx}\right)E_{x}+\left(J_{xy}-J_{yy}\right)E_{y}+R_{x}-R_{y}\right|}^{2}, \end{array}$$

(5) $$\begin{array}{cc} I_{4} & ={\left|J_{yx}E_{x}+J_{yy}E_{y}+R_{y}\right|}^{2}. \end{array}$$

To extract the complex optical field, we applied a Fast Fourier Transform (FFT) to the recorded holograms from $I_{1}$ to $I_{4}$, resulting in the corresponding spectra $F_{1}$, $F_{2}$, $F_{3}$, and $F_{4}$. Due to the presence of two orthogonally polarized reference beams, six distinct off-axis diffraction orders appear in the Fourier domain (Figure 3b).

By isolating each of these six diffraction orders in Fourier space and performing an inverse FFT, we retrieve the complex amplitude distributions of the interfering wavefronts. This operation is carried out with and without the sample inserted in the system. The fields recorded without the sample are used to compensate for system aberrations by dividing the respective complex amplitude distributions.

This method enables high-fidelity, single-shot retrieval of both phase and amplitude responses of anisotropic samples such as liquid crystals, providing a complete description of their local polarization-modulated behavior.

### 2.4. Limitations of the PHM System

The temporal resolution is primarily determined by the exposure time and the camera frame rate. In our case, the exposure time is 9 ms, and the frame rate is 73.29 Hz. These parameters are sufficient to resolve the formation dynamics of topological defects such as torons, whose characteristic transformation time is defined by the elastic relaxation time of the director field. This time can be estimated as follows:

(6) $$\tau_{\mathrm{elast}}=\frac{\gamma }{K}\cdot l^{2},$$

where γ is the rotational viscosity, *K* is the average elastic constant, and *l* is the relevant spatial scale. For E7 liquid crystal parameters, $\frac{\gamma }{K}$ is on the order of $10^{10}\;s\;m^{-2}$, resulting in an elastic relaxation time of tens of seconds for typical toron size of the order of tens of microns for used LC cells. Thus, the temporal resolution of our system is significantly faster than the intrinsic relaxation dynamics.

The spatial resolution is fundamentally constrained by the diffraction limit of the objective lens, estimated by 1.22λ/NA, which for our illumination wavelength and objective yields approximately 1 μm. In practice, the achievable resolution is also influenced by the fringe width in the interference pattern (about 5 pixels per fringe, corresponding to 0.3 μm) and the filtering window applied during Fourier processing, which defines the range of spatial frequencies retained in the reconstruction. In our case, the main spatial resolution is limited by the objective lens to approximately 1 μm, while other factors, including the fringe pattern and the filtering window used in Fourier processing, further constrain the practically achievable resolution to about 0.6–0.7 μm. It should be noted that the filtering does not improve the diffraction-limited resolution but restricts the highest spatial frequencies that can be reliably extracted. Additionally, the pixel size (or superpixel size) may further limit the resolution, particularly when the fringe period becomes comparable to the pixel dimension, leading to undersampling and aliasing.

For torons, the relevant spatial dimensions in all three directions correspond to the thickness of the liquid crystal film. The formation process can be interpreted as the minimization of the LC’s total free energy, which consists of the bulk elastic energy and the surface anchoring energy at the LC–glass substrate interfaces [43]. By varying the anchoring conditions (e.g., using different alignment materials), one can obtain other types of topological defect structures in addition to torons. On the other hand, increasing the film thickness reduces the relative contribution of the surface anchoring energy, complicating the controlled generation of torons. If torons are nevertheless formed in thicker LC films, their characteristic size adjusts accordingly but remains comparable to the film thickness.

## 3. Results

### 3.1. The Distributions of the Jones Matrix

The data processing yielded spatially resolved distributions of the Jones matrix coefficients, reconstructed at each camera pixel based on the optical response from the corresponding region of the LC sample. These include the amplitude components $|J_{xx}|$, $|J_{xy}|$, $|J_{yx}|$, and $|J_{yy}|$, and the phase components $∠J_{xx}$, $∠J_{xy}$, $∠J_{yx}$, and $∠J_{yy}$, reconstructed from polarization-resolved interferometric measurements. These distributions provide comprehensive insight into the anisotropic optical response and polarization modulation effects within the liquid crystal structure.

**Figure 4:**
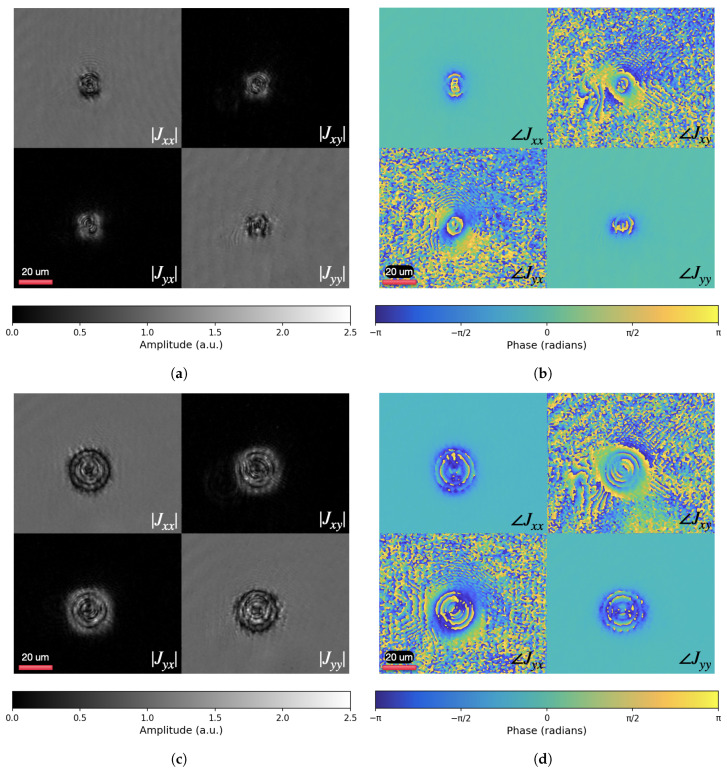
Jones matrix elements. Formation and optical response of a toron in a chiral nematic LC. (**a**) The amplitude distributions recorded approximately half a second after the start of focused laser irradiation, showing the initial stage of toron formation. (**b**) Corresponding phase distributions at the same moment. (**c**) The amplitude distributions of a metastable toron recorded after 4.5 s following irradiation cessation. (**d**) Corresponding phase distributions of the metastable structure.

Figure 4a shows the amplitude distributions of the Jones matrix coefficients $\left|J_{xx}\right|$, $\left|J_{xy}\right|$, $\left|J_{yx}\right|$, and $\left|J_{yy}\right|$ during the initial stage of toron formation, recorded approximately half a second after the onset of focused laser irradiation. In contrast, Figure 4c presents the corresponding amplitude maps for a metastable toron configuration captured after 4.5 s following irradiation cessation. Although this time point may not fully correspond to a thermodynamically relaxed state, it reflects a metastable configuration in which the main features of the toron are present. These amplitude distributions provide insight into the local anisotropy and optical response of the LC sample. The components $\left|J_{xy}\right|$ and $\left|J_{yx}\right|$, in particular, highlight regions where the polarization state of transmitted light is altered due to molecular reorientation. In areas surrounding the toron core, where these off-diagonal elements exhibit significant intensity, the LC molecules are misaligned with respect to the optical axis, indicating a strongly birefringent and anisotropic state. In contrast, the dark zones in $\left|J_{xy}\right|$ and $\left|J_{yx}\right|$ correspond to spatial regions where the Jones matrix approximates an identity form $\left[\begin{array}{cc} 1 & 0\\ 0 & 1 \end{array}\right]$, implying that the transmitted light preserves its polarization. This behavior is characteristic of homeotropic (perpendicularly aligned) LC regions, which act as optically isotropic layers. The transition from this uniform background to the structured, anisotropic toron core reflects the emergence of complex director fields responsible for modulating the polarization state of light. The amplitude scale in Figure 4a,c extends beyond unity, reaching values of up to 2.5. This is due to the fact that the reconstructed amplitude maps reflect energy redistribution effects rather than pure transmittance. Since torons are inherently three-dimensional structures and the reconstruction is performed in a single image plane, the measured field captures interference effects and local phase retardation contributions accumulated along the 10 mμ LC cell thickness. As a result, localized constructive interference can lead to effective amplitude values exceeding 1.

The phase distributions of the Jones matrix coefficients $∠J_{xx}$, $∠J_{xy}$, $∠J_{yx}$, and $∠J_{yy}$, shown in Figure 4b,d, provide information on the local optical path differences across the LC sample. These maps capture the spatial modulation of the phase delay imparted by the anisotropic structure of the chiral nematic liquid crystal. In the region of the toron, one can observe continuous phase wrapping and localized phase singularities, which result from the complex three-dimensional director configuration. Importantly, the toron is not a singular defect, such as a point defect or disclination line, but a well-defined topological soliton that incorporates within its structure two hyperbolic point defects (hedgehogs) and a twisted director field [5]. Structured phase profiles, especially visible in the non-diagonal elements $∠J_{xy}$ and $∠J_{yx}$, reflect the modulation of the phase dependent on the polarization caused by the toron. The surrounding uniform homeotropic LC background exhibits flat phase distributions consistent with optically isotropic behavior. This contrast highlights the localized optical complexity of the toron and its impact on the polarization-resolved optical field.

Overall, toron generation required less than one second of light illumination under the given laser irradiation conditions. The formation process occurs through localized heating by focused femtosecond laser pulses at an average intensity of approximately 255 kW/cm^2^. For a focused beam diameter of 5 µm and an actual exposure time of approximately 0.93 s, this results in a total delivered energy per unit area of approximately 237 kJ/cm^2^. This level of localized heating induces a transient transition to the isotropic phase, followed by rapid cooling of the LC into the mesophase after the laser is switched off, and then relaxation of the director field into a metastable toron configuration. This is evidenced by the evolving amplitude and phase maps. The Supplementary Video (Toron_formation.mp4) captures the complete dynamics, lasting about 4s, and displays synchronized amplitude and phase distributions of the evolving structure. Upon laser activation (0.39 s), the phase map reveals an initial localized spot, indicating the onset of director field distortion and the start of toron formation. Subsequently (0.43 s), the amplitude distribution develops a characteristic Maltese cross pattern, signifying the umbilic-like director field configuration. From 0.45 s onward, the formation of an isotropic core becomes apparent, surrounded by a growing thermally distorted region. At 1.32 s, the laser is switched off, and the system begins to transform and undergo cooling, during which the isotropic region transitions to the mesophase. This relaxation process ultimately leads to the stable toron structure shown in Figure 1.

It should be noted that we used the left-handed chiral dopant S811 to form the chiral nematic LC. From the point of view of toron formation, there is no fundamental reason to expect a different formation mechanism if the right-handed chiral dopant R811 was used instead. However, due to the opposite handedness of the induced cholesteric helix, one would expect differences in the resulting polarization-resolved interferograms and the corresponding Jones matrix distributions since the twisting direction of the double-helix cylinder in the midplane would be reversed. Nevertheless, this work represents a first step towards applying digital holography to study such topological structures, and comparative experiments using oppositely oriented chiral dopants will be considered in future studies.

This analysis demonstrates that polarization holography enables the precise characterization of topological structures in chiral nematic liquid crystals, providing detailed insights into their optical properties and stability. The quantitative visualization of these structures through both phase and amplitude channels enhances our understanding of defect dynamics and could support future developments in tunable photonic devices and topological optical elements.

### 3.2. Torons for Spin–Orbit Conversion

The findings of this study not only validate the efficacy of the proposed PHM technique for analyzing topological structures in liquid crystals but also demonstrate the functional versatility of these structures. As an example, torons behave similarly to a q-plate, which is an element capable of generating vortex beams [44,45,46]. When a left-circularly polarized (LCP) beam passes through the toron followed by a quarter-wave plate (QWP), the resulting vector field can be described by the following:

(7) $$\tilde{E}=\frac{1}{2\sqrt{2}}e^{-i\pi /4}\left(\begin{array}{cc} 1+i & i-1\\ i-1 & 1+i \end{array}\right)\left(\begin{array}{cc} J_{xx} & J_{xy}\\ J_{yx} & J_{yy} \end{array}\right)\left(\begin{array}{c} 1\\ i \end{array}\right),$$

where

- $\frac{1}{2\sqrt{2}}e^{-i\pi /4}\left(\begin{array}{cc} 1+i & i-1\\ i-1 & 1+i \end{array}\right)$ is the QWP;
- $\left(\begin{array}{c} 1\\ i \end{array}\right)$ is the LCP beam.

The spin–orbit conversion capability of torons is demonstrated through Jones matrix-based numerical modeling of the transmitted optical field.

**Figure 5:**
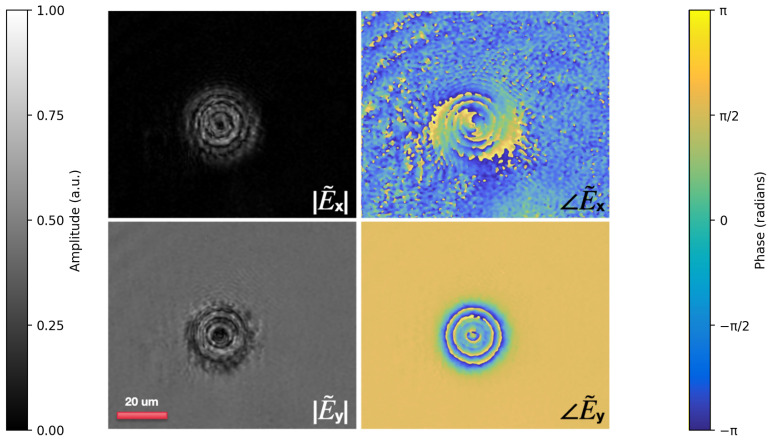
Amplitude–phase profiles of the vector field after LCP beam transmission through a toron and QWP. Amplitude (|${\tilde{E}}_{x}$|, |${\tilde{E}}_{y}$|) and phase ($∠{\tilde{E}}_{x}$, $∠{\tilde{E}}_{y}$) distributions of the vector field generated by an LCP plane wave transmitted through a toron and QWP. |${\tilde{E}}_{x}$| and |${\tilde{E}}_{y}$| are the amplitudes of the horizontal and vertical field components; $∠{\tilde{E}}_{x}$ and $∠{\tilde{E}}_{y}$ depict their phase profiles.

As shown in Figure 5, the horizontal field component (**${\tilde{E}}_{x}$**) exhibits a vortex beam with topological charge +2, while the vertical component (**${\tilde{E}}_{y}$**) displays a lensing effect. This dual functionality vortex generation in one polarization component and focusing in the other confirms the toron’s role as a multifunctional optical element.

These results match both the Jones matrix predictions (Equation (7)) and the experimental findings for cholesteric spherulites [47], where a similar polarization-controlled modulation of orbital angular momentum was observed. This agreement demonstrates that torons can effectively emulate q-plate behavior for spin–orbit conversion, highlighting their potential for dynamic photonic applications requiring control of light’s vectorial properties.

## 4. Discussion

Due to the intrinsically three-dimensional and spatially varying nature of the director field in topological solitons and other orientational structures in liquid crystals [4,48], it is fundamentally impossible to fully reconstruct it using only two-dimensional optical microscopy under crossed polarizers. The most established techniques for three-dimensional director field reconstruction include fluorescence confocal polarizing microscopy and various non-linear optical microscopy approaches [49]. These methods typically capture the director field in a metastable configuration rather than during its dynamic evolution. An alternative approach involves numerical modeling, where the initial configuration is approximated and then relaxed by minimizing the elastic free energy of the LC medium [5,50]. Although this enables 3D reconstruction, it still does not provide real-time insight into the formation dynamics of orientational structures.

To study the temporal evolution of the director field during the formation of orientational structures, several experimental strategies have been proposed. One commonly used approach involves stabilizing the LC in intermediate states via photopolymerization. These “frozen” configurations can subsequently be examined using advanced techniques such as electron microscopy [51]. However, photopolymerization may introduce distortions to the original LC configuration, while fluorescent dyes often absorb in the UV or blue spectral range, potentially modifying the optical properties of the system [52]. In addition, this approach is labor-intensive, as it requires capturing and analyzing multiple intermediate states at different time points during the formation process.

The implementation of the PHM system offers several practical advantages for the quantitative analysis of anisotropic and topologically structured optical media. Its configuration, combined with a polarization-sensitive camera, allows the separation of interference signals without the need for polarization rotation optics.

In terms of torons, because of their ability to modulate the polarization state of transmitted light, they are promising candidates for use in integrated photonic circuits. Such topological LC components are actively being explored for compact and reconfigurable light control in next-generation optical systems [53]. This integrated approach bridges fundamental LC topology and applied photonics. Future work could explore topological structures, in particular torons, as an alternative to conventional q-plates [44,45].

## 5. Conclusions

In this work, we presented an integrated approach to studying dynamic topological phenomena in chiral nematic liquid crystals by combining real-time optical probing with a custom-built polarization holographic microscope. We applied this technique to investigate the real-time formation of torons and demonstrated how these solitonic structures modulate transmitted light and act as functional optical elements, with potential applications in polarization shaping and spin–orbit light conversion. The implementation of the PHM not only advances the methodology for observing light–matter interactions in anisotropic systems but also provides a practical tool for studying director field evolution during structure formation.

Overall, this study establishes three key contributions. Specifically, it provides the following:

- A demonstration of the spin–orbit conversion functionality of torons, which act similarly to q-plates by modulating the polarization and phase structure of transmitted light;
- A real-time polarization-resolved interferometric system for monitoring their evolution;
- The capability to track the formation and dynamics of topological configurations with high temporal and spatial resolution.

These developments pave the way for the integration of tunable LC-based elements into next-generation optical platforms, including adaptive sensors, reconfigurable metasurfaces, and polarization-controlled laser systems.

## Data Availability

The data presented in this study are available on request from the corresponding author. The data are not publicly available due to their inclusion in ongoing research.

## Supplementary Materials

The following supporting information can be downloaded at https://www.mdpi.com/article/10.3390/polym17131849/s1. The video (Toron_formation.mp4) is slowed down six times and shows the complete formation dynamics of a toron, visualized using synchronized amplitude (left) and phase (right) maps of the Jones matrix coefficients ($\left|J_{xx}\right|$, $\left|J_{xy}\right|$, $\left|J_{yx}\right|$, and $\left|J_{yy}\right|$; $∠J_{xx}$, $∠J_{xy}$, $∠J_{yx}$, and $∠J_{yy}$). The video captures the onset of director field distortion under focused femtosecond IR laser irradiation (0.39 s), the appearance of the characteristic Maltese cross pattern (0.43 s), the formation of an isotropic core and the surrounding thermally distorted region (0.45 s), and, after laser termination at 1.32 s, the subsequent cooling and relaxation of the director field into the metastable toron configuration.

## Abbreviations

The following abbreviations are used in this manuscript.

BS	Beam Splitter
DM	Dichroic Mirror
FFT	Fast Fourier Transform
IR	Infrared
LC	Liquid Crystal
LCP	Left-Circularly Polarized
M	Mirror
OAM	Orbital Angular Momentum
OBJ	Microscope Objective
P	Prism
PBS	Polarizing Beam Splitter
PHM	Polarization Holographic Microscope
PS	Polarizing Sensor
QWP	Quarter-Wave Plate
UV	Ultraviolet
WP	Wollaston Prism
